# ‘Better medicines for children’ within the Integrated Management of Childhood Illness framework: a qualitative inquiry in Uganda

**DOI:** 10.1186/s40545-016-0071-9

**Published:** 2016-06-07

**Authors:** Xavier Nsabagasani, Japer Ogwal-Okeng, Ebba Holme Hansen, Anthony Mbonye, Herbert Muyinda, Freddie Ssengooba

**Affiliations:** Child Health and Development Center, College of Health Sciences, Makerere University, Kampala, Uganda; Department of Pharmacology and Therapeutics, Gulu University, Gulu, Uganda; Section for Social and Clinical Pharmacy, Department of Pharmacy, Faculty of Health and Medical Sciences, University of Copenhagen, Copenhagen, Denmark; Department of Community Health, Ministry of Health Uganda, Kampala, Uganda; Department of Health, Uganda Christian University, Mukono, Uganda; School of Public Health, College of Health Sciences, Makerere University, Kampala, Uganda

**Keywords:** IMCI, Better medicines for children, Dosage formulations and Uganda

## Abstract

**Background:**

The Integrated Management of Childhood Illnesses is the main approach for treating children in more than 100 low income countries worldwide. In 2007, the World Health Assembly urged countries to integrate ‘better medicines for children’ into their essential medicines lists and treatment guidelines. WHO regularly provides generic algorithms for IMCI and publishes the Model Essential Medicines List with child-friendly medicines based on new evidence for member countries to adopt. However, the status of ‘better medicines for children’ within the Integrated Management of Childhood Illnesses approach in Uganda has not been studied.

**Methods:**

Qualitative interviews were conducted with: two officials from the ministry of health; two district health officials and, 22 health workers from public health facilities. Interview transcripts were manually analyzed for manifest and latent content.

**Results:**

Child-appropriate dosage formulations were not included in the package for the Integrated Management of Childhood Illnesses and ministry officials attributed this to resource constraints and lack of initial guidance from the World Health Organization. Underfunding reportedly undercut efforts to: orient health workers; do support supervision and update treatment guidelines to reflect ‘better medicines for children’. Health workers reported difficulties in administering tablets and capsules to under-five children and that’s why they preferred liquid oral dosage formulations, suppositories and injections.

**Conclusions:**

The IMCI strategy in Uganda was not revised to reflect child-appropriate dosage formulations – a missed opportunity for improving the quality of management of childhood illnesses. Funding was an obstacle to the integration of child-appropriate dosage formulations. Ministry of health should prioritize funding for the Integrated Management of Childhood Illnesses and revising the Essential Medicines and Health Supplies List of Uganda, the Uganda Clinical Guidelines and, the Treatment Charts for the Integrated Management of Childhood Illnesses to reflect child-appropriate dosage formulations.

## Background

Globally, an estimated 8.1 million children under-five die every year due to conditions that can be prevented or treated with evidence-based medicines [1]. In 1995, the World Health Organization (WHO) and United Nations Children’s Emergency Fund (UNICEF) introduced the Integrated Management of Childhood Illnesses (IMCI) strategy to improve case management skills of health workers to diagnose and treat the major childhood illnesses. IMCI was meant to address three key elements. The first element was to develop and provide IMCI guidelines to enable health workers improve case management. Therefore, WHO regularly provides generic algorithms for IMCI to be adopted by countries following their epidemiological situation. WHO also supports countries in the selection of essential medicines for children by publishing the Model Essential Medicines List for children [2]. The second element was to build the capacity of health workers through training and support supervision to enable them use the provided IMCI algorithms to identify and treat illness. The third element was to strengthen the health systems so as to provide an environment and facilities conducive for managing children illnesses. This element covers ensuring that there is availability of priority medicines for children. Therefore, UNICEF recommends that governments should improve their health systems to ensure that the essential drugs are available [3]. Some studies have concluded that availability of medicines is a key condition for the success of IMCI [2–6].

In 2007, the 60th World Health Assembly made a resolution that urged member countries to include ‘better medicines for children’ in their essential medicines lists and clinical guidelines [4]. Following this resolution, later in 2007 WHO and partners launched the ‘make medicines child size’ campaign to create more awareness about child-appropriate dosage formulations [5]. Hence, it was sensible to integrate child-appropriate dosage formulations into IMCI, a strategy that is used to manage sick children in more than 100 countries worldwide. Uganda was among the first countries worldwide to adopt and scale up IMCI. According to personal communication with a ministry of health official, after piloting of the strategy in 1995, IMCI was adopted in 1997 as a national strategy for the management of the common childhood illnesses. The first phase of implementation of IMCI included adaptation of the globally recommended IMCI guidelines to the Ugandan setting and consequent training of the health workers in selected districts. With support from the United States Agency for International Development (USAID), the IMCI approach was first pilot tested in ten districts in 1996 and a 22 member national task force was established to coordinate the IMCI activities. In 1997, the IMCI approach was scaled up nationally.

According to the Uganda Government Heath Sector Strategic and Investment Plan III (HSSIP III), IMCI was recognized as the main approach for treating children in Uganda [6]. Despite that recognition, there are still gaps in policy provisions for child-appropriate dosage formulations and so far there has been no formal adoption of these formulations as a policy [7, 8]. Furthermore, there is still low availability and utilization of the WHO recommended lifesaving priority medicines for children in the public health facilities [9].

Despite these gaps, the perspectives: of administrators at the MoH; district health officials; and, health workers’ practices and experiences of treating children within the IMCI approach using child-appropriate dosage formulations have not been explored. This paper is premised on the understanding that IMCI should be the vehicle for integrating child-appropriate dosage formulations in those countries where it is being implemented. It is therefore critical to assess the extent to which IMCI implementation at the facility level reflects the WHA resolution of ‘better medicines for children’. This paper explores the perspectives of officials from MoH and the district health department about the integration of ‘better medicines for children’ into the IMCI approach. At the facility level, the paper explores health workers’ knowledge and perspectives about child-appropriate dosage formulations, their practices and experiences in the management of childhood illnesses in Uganda using IMCI approach.

## Methods

### Study design

The study derives from the 60th WHA resolution of ‘better medicines for children’. Therefore, the focus of the study was about the level of integration and application of the ‘better medicines for children’ concept into IMCI, the main framework for managing childhood illnesses in Uganda. The study design was also based on the premise that child-appropriate dosage formulations such as dispersible tablets, granules, sprinkles and pellets are the best for improving prescription, dispensing and administration of medicines to the under-five population [10]. In this paper, child-appropriate dosage formulations refer to medicines that are easily adjusted to reflect size, strengths, state of development, and condition of a child [10]. WHO recommended dispersible, scored tablets or the equivalent of the flexible solid oral dosage formulations as the most effective for children [1, 11]. This study applied these concepts as basis for the formulation of the analytical framework, the data collection methods and the tools that were used.

The analytical framework of the study draws from debates about policy processes and factors that influence success of implementation of policy and its sustainability [12–14]. Tansella &Thornicroft (2009) highlighted these factors in relation to how to accelerate the transfer of discoveries in health sciences into implementation of evidence based routine clinical practice. Tansella &Thornicroft articulated three phases of implementation of evidence based clinical practices: adoption in principle; early implementation and, persistence of implementation [12]. This framework helps to explain the process and context of IMCI adoption, implementation and its implication on the adoption of child-appropriate dosage formulations in a low income country Uganda. In this study, facilitators and barriers to the implementation of the IMCI program are considered. A multi-level analysis of perspectives is used and covers the national, district and facility levels.

### Study setting

The study was conducted between July and August 2012 in Uganda, a landlocked low income country in the East African region. In Uganda, IMCI is the main approach for diagnosing, dispensing and administering medicines to treat childhood illnesses. Uganda was also among the first UN member states in sub-Saharan Africa to implement IMCI as a national program since 1995 [6]. An IMCI section was included into both the Uganda Clinical Guidelines 2010 and 2012 versions [15, 16].

In Uganda, procurement of medicines for public health facilities is under National Medical Stores (NMS) which distributes medicines provided in the Essential Medicines and Health Supplies List of Uganda (EMHSLU) using a push system. By the time of the study, the latest version of EMHSLU was that of 2012. Other medicine procurement and distribution channels included: the Joint Medical Stores (JMS)-an umbrella organization for the Uganda Catholic Medical Bureau (UCMB) and Uganda Protestant Medical Bureau and, a private sector procurement agent called Medicine Access Uganda Limited (MAUL). Some medicines are also accessed with direct support of donors and are channeled through some development partners: the Uganda Health Marketing Group (UHMG); partners funded by the President’s Emergency Plan for AIDS Relief (PEPFAR); Malaria Consortium (MC) and, the United Nations (UN) agencies including UNICEF, the Joint United Nations Programme on HIV and AIDS (UNAIDS) and WHO.

The Ugandan public health care system is highly decentralized with districts and health sub-districts playing a key role in the administration of the delivery of health services. There is a hierarchy of public health facilities starting with the lowest service delivery point being at the village level now referred to as Health Center One (HCI) through to Health Center four (HCIV) at a county level. Above these there is , the district hospitals, regional and national referral hospitals at the top [6]. At HCI, services are provided by volunteers referred to as Village Health Team (VHTs) who do not have formal health training. In 2010, the VHTs were given an additional role of distributing medicines for the integrated community case management (ICCM) of childhood illnesses. The Health Center two (HCII) facilities are largely managed by Nursing Assistants who have minimal training in the medical field. In most of the rural facilities in Uganda, Nursing Assistants play a significant role in treating children. Health Center three (HCIII) facilities are supposed to be served by clinical officers, nurses, midwives and nursing assistants. In reality, due to staffing challenges at this level, Nursing Assistants still constitute the majority of the health workers. At Health Center Four (HCIV) level, in addition to the cadre of staff stated above, there should be at least a medical doctor. At the district hospital and above, there are both general doctors and specialists in addition to other cadres of health staff. In case of children, the specialists are pediatricians.

### Study respondents

Qualitative in-depth interviews were held with: the Ministry of Health officials; the district health team and, health workers from the public health facilities. Health facilities included one national referral hospital and one regional referral hospital and sixteen lower level rural based health facilities in Jinja district. In Uganda, the concept of health workers refers to those people working in the health system with the qualifications recognized by government. Rural based health facilities were lower levels facilities in the rural areas of Jinja district. These included HCIIs, HCIIIs and HCIVs. Table 1 shows the categories of respondents and the themes included in the interview guide for each category.

**Table 1:** Respondents and the thematic areas covered

Respondent	Number	Main themes covered
MoH Program mangers officers)	2	• The historical context and the process of IMCI implementation • What worked and what did not work with IMCI and child-appropriate dosage formulation adoption • The level of integration of the child-appropriate dosage formulation into IMCI approach • Achievements and limitation of the integration
The district health team	2	• The historical context and the process of IMCI implementation • Perspective of the child-appropriate dosage formulations • Experiences of implementation of IMCI and child-appropriate dosage formulations under a decentralized arrangement • Financing, support supervision and refresher training
Pediatricians from Mulago hospital	5	• Practices about using child-appropriate dosage formulations to treat children under 5 years • Available information about child-appropriate dosage formulations • Coping mechanisms when there are no child-appropriate dosage formulations
Pediatrician from Jinja hospital	1	• Practices about using child-appropriate dosage formulations to treat children under 5 years • Available information about child-appropriate dosage formulations • coping mechanisms when there are no child-appropriate dosage formulations
Lower level rural based health workers	16	• Their perspectives and practices about using child-appropriate dosage formulations to treat children under 5 years within the IMCI framework • Available information about child-appropriate dosage formulations and the treatment protocols • Coping mechanisms when there are no child-appropriate dosage formulations

At the Ministry of Health level, two officials from the child health division were interviewed. The child health division was behind the adaption and scaling up IMCI implementation in the country. The division also spearheaded the adaptation of the WHO IMCI algorithm, initial training and support supervision of health workers. Two health officials from Jinja district in eastern Uganda were purposively selected to participate in the study. The district health officials had been part of the early implementation of IMCI in the district.

At the facility level, five pediatricians were drawn from Mulago, the biggest national referral hospital located in the capital city Kampala. The national referral hospital is a teaching hospital for the Makerere College of Health Sciences. One pediatrician was drawn from Jinja hospital, the biggest eastern regional referral hospital located in Jinja town. Despite, the huge pediatric patient load in the regional referral hospital, there are only two pediatricians. Sixteen lower level health workers were drawn from 16 rural based health facilities in Jinja district.

All the respondents were purposively selected based on experiences and current responsibilities about the implementation of the IMCI strategy. At the facility level, those found treating children at the time of the visit were automatically selected for interviews. All interviews were held in privacy and in venues preferred by the respondents. Where it was felt that information saturation was realized we stopped interviewing more of that category of respondents. There were 16 health workers from the rural based health facilities. This number was high because we wanted to get information from the diverse categories (clinical officers, nurses, midwives and nursing assistants) who represented different levels of healthcare.

Jinja district has a population of more than 468,256 people and of these 299,109 (64 %) live in rural areas [17]. Administratively, the district is subdivided into seven rural sub-counties, one town council and three divisions in the municipality. Like the rest of the country, there is a hierarchy of health facilities ranging from Health Centre II to the regional referral hospital as described elsewhere [9]. Jinja district has a total of 69 health facilities, 49 of which are public, seventeen are run by NGOs and three are institutional (army, police and prisons). The major causes of child morbidity and mortality in the area include malaria (70 %), acute respiratory infections (16 %) and diarrheal diseases (32 %) [18]. Jinja is also a home of the neglected tropical disease of schistomiasis which affects both adults and children.

## Data collection

Qualitative in-depth interviews were performed using an unstructured thematic interview guide. For each category of respondents, as earlier indicated, the key themes addressed are provided in Table 1. Data was collected by the first Author (a social scientist) and was assisted by a Research Assistant who is also a social scientist. All the interviews were conducted in English by the first author. On average the interviews lasted between 40 and 60 min.

## Data analysis

Interviews were digitally recorded and later transcribed verbatim, printed and carefully read. The codes were then applied to the scripts. Data analysis was conducted manually using a thematic manifest and latent content analysis [19]. A coding sheet with the key themes was developed and later applied to the transcribed text. Deductive meanings that were theory guided were adopted from our pre-conceived abstractions and compared with the primary data. This was based on the understanding that category names and related codes can be generated from the already existing concepts, literature and the phrases used by the informants themselves [20].

## Results

The study explored perspectives about the status of child-appropriate dosage formulations within the IMCI approach at three levels in Uganda: the central ministry level; the district level and the health facility level. The key themes that emerged were as follows:

- Lacking of funding cut across all the three levels and affected implementation of IMCI
- At the national level, the IMCI strategy had not been revised to include child-appropriate dosage formulations
- At the district level, the decentralized IMCI activities were not being implemented and this weakened the efforts to integrate the child-appropriate dosage formulation component. Ironically, re-centralization of medicine procurement and distribution to the National Medical Stores (NMS) reversed district’s discretion to procure child-friendly medicines
- At the facility level, health workers’ faced challenges of prescribing, dispensing and administering tablets and capsules to children. They remodeled ways to ensure that children received appropriate treatment. Lower level cadres of rural based health workers did not have sufficient information about the new treatment policies that might have included child-appropriate dosage formulations

## Resource constraints

The predominant challenge to the integration of child-appropriate dosage formulations was the insufficient funding of IMCI activities at the national, district and facility levels. Table 2 shows that at the national level, resource constraints affected planning, development of relevant policy reforms and budgeting that in turn affected the integration of child-appropriate dosage formulations into the IMCI framework. This in turn had a spillover negative effect on the implementation of IMCI at the lower levels. MoH officials reported that due to lack of funding, it was not possible to revise the IMCI treatment charts to reflect child-appropriate dosage formulations. During the late implementation phase of IMCI, the project resources were already exhausted and when government took over, it failed to sustain most of the activities.

**Table 2:** The process of IMCI Development and implementation landmarks in Uganda

Levels	Implementation phases		
	Adoption phase	Early implementation	Situation at the time of the study
National	In 1995 IMCI selected as the main approach for Uganda. A 22- member working group was established for planning, training and adaptation. IMCI algorithm was adopted to fit the national health policy and treatment guidelines	By 1998 there was a national expansion plan with a pool of 250 trainers, 10 zonal teams to supervise IMCI implementation. Donors: World Bank, USAID, UNICEF and WHO put in resources for early implementation of IMCI. By 2003 all the districts in Uganda had been trained in IMCI	Budget constraints and lack of information about ‘better medicnes for children’ meant limited national level reflections about policies, plans, budgets and guidelines for child-appropriate dosage formulations. No resources for the sustainability of IMCI in terms of refresher trainings, revision of job aids and support supervision. The decentralization of IMCI to districts minimized the central MoH role both technically and financially.
Local (district)	Districts health officials were prepared in terms of training about IMCI.	Districts were prepared in terms of training about IMCI.	Resources were not available for the districts. Districts were getting minimal support from the ministry in form of PHC grants which covered a little on support supervision. Therefore, the districts were not prepared for training health workers on child-appropriate dosage formulations
Facility/individual levels	Health workers were sensitized on the new approach of IMCI.	Health workers were trained on the new approach of IMCI for 3 weeks.	Health workers were no longer receiving refresher training and support supervision for IMCI related activities from the district health team because districts did not have the financial capacity to do so.

*At the moment we do not have money for continuing some of the IMCI activities the way we are supposed to. Donors have shifted ICCM. We have adopted the IMCI approach as a routine but we do not have needed resources to sustain some of the core activities including revision of guidelines and refresher training (MoH, official).*

MoH officials perceived some of the child-appropriate dosage formulations recommended by WHO such as dispersible tablets as a luxury for the already financially hard-pressed ministry. MoH officials were also aware of the fact that there were no funds for the decentralized IMCI activities. Districts were expected to use the Primary Health Care (PHC) grants to implement the decentralized IMCI activities and development partners were expected to supplement. However, the PHC grants were not enough to cater for all the decentralized IMCI activities and other already earmarked activities. Jinja district did not get support for IMCI from development partners.

*As a ministry, we no longer handle IMCI refresher training. This function was decentralized to the districts that have to develop a budget to cater for the refresher training and support supervision. A few districts have been lucky and some partners like STRIDES are supporting IMCI. As of now we do not know what is going on in the districts except where the partners are operating now (Ministry of Health Official).*

MoH was also aware of the human resource challenges especially understaffing and limited number of qualified health personnel especially in the rural areas. Such staff needed adequate refresher training and support supervision to acquaint them with new information about the global and national treatment policies for children.

### Failure to revise the IMCI treatment charts

Despite of the fact that WHO consistently revises the IMCI algorithm to accommodate new evidence based medicine, MoH officials admitted that it has not been possible to adapt new WHO IMCI algorithms to the Ugandan setting due to funding challenges as already indicated. Other than the WHA recommendation of better medicines for children, there other national initiatives such as changes in malaria treatment policies which were not communicated to the lower cadre staff. All these were not reflected in the IMCI treatment charts and this resulted into confusion especially among the lower level rural based health workers who entirely depend on IMCI treatment charts to diagnose and prescribe medicine to children.

### Lack of global and national level efforts to initiate the integration

Unlike the other initiatives such as malaria treatment policy, WHO did not initiate national discussions about child-appropriate dosage formulation and how they would be integrated into the IMCI framework. MoH expected external support especially from WHO and UNICEF to initiate and finance the integration of child-appropriate dosage formulations.

*We did not see any justification for just introducing child-appropriate dosage formulations into the IMCI approach without any guidance from the World Health Organization. There were gaps in the information about the child-appropriate dosage formulations concept especially regarding where the medicines could be procured and at what prices. (Ministry of Health official).*

On the other hand, donors had stopped funding facility based IMCI by the time the resolution of 'better medicines for children' was made. The MoH officials recounted how they had advised donors to continue to support the facility based IMCI including procurement of medicines for which donors did not comply. The discussions about child-friendly medicines only gained momentum in 2012 after the UN declaration of life- saving priority medicines for children. Both government and donors agreed to include into the mainstream health service delivery system, lifesaving for children. This was a good opportunity since all the medicines in the list of priority lifesaving medicnes for children were recommended in child-appropriate dosage formulations. In spite of this new emphasis, MoH officials also confessed that it would take time before these medicines could be accessed as there would be need to revise the EMHSLU, UCG and IMCI treatment charts to reflect ‘better medicines for children’.

## Perspectives about child-appropriate dosage formulations

Table 3 highlights the context and explanations by the respondents at different levels about status quo of child-appropriate dosage formulations within the IMCI framework in Uganda. The table highlights the perspectives in the wider context of the three different levels (ministry, district and facility) and how this affected the integration of child-appropriate dosage formulations into the IMCI framework. Although there were some areas of convergence, the perspectives are presented according to the specific respondents: MoH officials, district health officials, pediatricians and lower level rural based health workers.

**Table 3:** Perspectives of respondents about IMCI and child-appropriate dosage formulations

Actors	The context	The Point of view of the actors
MoH officials	• Budget constraints and lack of information about ‘better medicines for children’ meant limited national level reflections about policies, revision of guidelines, budgets and guidelines for child-appropriate dosage formulations • MoH decentralized refresher training and support supervision for IMCI for the districts and their development partners	• Lack of global initiatives to support the resolution of ‘better medicines for children’ at the national level and challenges of sustainable financing for IMCI implementation • When IMCI funding reduced and MoH could no longer provide oversight and refresher training for IMCI and these roles were left for local governments under decentralized services. However, MoH continues to acknowledge the fact that there were no sufficient resources for implementing IMCI under the decentralized arrangement • Concerns that donors and development partners had shifted their goals to fund the community based ICCM program completely leaving out support for IMCI at the facility level • Since the markets and prices for some of the child-appropriate dosage formulations such as sprinkles, pellets and dispersible tablet were not known. They were perceived to be expensive and therefore not affordable by the ministry. Other well-known child-friendly formulations such as syrups were too costly and moreover difficult to handle and store in a low income setting of Uganda. They were deleted from the EMHSLU 2012 due to these reasons.
The district health officials	• Underfunding of the decentralized responsibilities in the health sector • Donors had shifted interest to ICCM and abandoned IMCI	• Jinja district was not ready for the decentralized roles of conducting IMCI refresher training and support supervision due to financial constraints. They were concerned about lack of revised IMCI treatment charts. Centralization of medical supplies to NMS has deprived the district the opportunities to decide on the procurement of the right medicines for children
Pediatricians	• Pediatricians are limited and are only available in hospitals • Pediatricians are supposed to see children who are referred to them by the lower level health workers	• IMCI is a strategy to be used by the lower cadre staff. At the moment the approach is not robust enough to integrate new treatment changes • The IMCI strategy would benefit from improved IMCI guidelines that would address evidence based and child-appropriate dosage formulations • The Uganda Clinical Guidelines are insufficient and pediatricians were reportedly referring to other guidelines such as BNF and the internet to determine treatment. Prescribe some syrup such as amoxicillin syrup and other child-friendly syrups available in the private sector. Had an idea about dispersible tablets but did not use them because they were not available.
Lower cadre health workers (enrolled nurses and nursing assistants)	Cognitive Barriers, using outdated IMCI charts, limited interaction with the MoH officials in terms of support supervision	• Need refresher training on IMCI, updated guidelines and evidence based medicines • Need support supervision to enable them improve their skills • Some nursing assistants said that they did not know that the treatment policy for malaria had changed

## Perspectives from Ministry of Health

The concept of child-appropriate dosage formulations in Uganda centered on liquid oral dosage formulations (syrups) which were deleted from the EMHSLU 2012 and were for the interim replaced with tablets for adults that were extrapolated for children. The reasons for deleting syrups included high prices and handling challenges. The deleted syrups were no longer being supplied in the public health facilities.

*We usually find a lot of problems with these syrups. They are messy, expensive, and difficult to handle during transportation. They are had to store especially in remote areas where there is no power for refrigeration (Ministry of Health official)*

When asked about other child-appropriate dosage formulations such as pellets, sprinkles and dispersible tablets, MoH officials stated that lack of market information and high cost of these formulations repulsed efforts to integrate them into the IMCI approach. MoH officials generally perceived some of these formulations to be expensive and this affected their efforts to integrate them into the EMHSLU.

## The perspectives of the district health officials

The district officials’ explanations of the failure to integrate child-appropriate dosage formulations were consistent with those of the MoH. Lack of funds purportedly affected implementation of the decentralized IMCI activities. While the district managers were aware of their decentralized roles in relation to IMCI training and support supervision of facilities, they were not capable of carrying out the responsibilities due to lack of resources as indicated in Table 3. Under the decentralized arrangement, there was a challenge of understaffing due to the failure of local government to attract qualified health workers especially in the rural health facilities. Consequently, the majority of health workers seeing children in these facilities were mainly nurses and nursing assistants. Due to their limited medical training and limited access to information, this category of staff need constant on- job training and job aids to help them understand new information. According to the district officials, this was not happening.

*We are expected to carry out refresher training for different things including IMCI. The PHC grants that we receive are not enough for the many obligations. So we can only do a little in support supervision which also covers a wide range of other things. Refresher training for IMCI requires a lot of money which is not available. We are looking forward for some external support* (*District Health official, Jinja District)*.

District officials also underlined challenges of recentralizing procurement and distribution of medicine supplies to NMS which was supplying medicines that were not necessarily corresponding to the priorities of the district and health facilities. There was a general feeling that districts have no say about the medicines that are procured by NMS compared to before when the districts had the discretion to purchase the medicines they wanted. District official wondered why important medicines for children such as syrups were being left out by NMS.

*We used to have important syrups to treat malaria and pneumonia especially among the under-five children and now there are no more because we depend on NMS for drug supplies. NMS no longer supplies syrups. They supply tablets instead. It is now difficult for our caregivers to administer tablets to children. There are so many associated problems, dividing the tablets affects dosing, children refuse to swallow the medicines and if they do so they can easily vomit (District Health Official, Jinja district).*

Like the ministry, the district officials accentuated the limitation of unrevised IMCI treatment chart as a reference material especially for the nursing assistants who are not sufficiently medically trained but nevertheless were treating children in most of the low level rural based health facilities.

*In most cases our health workers use guess work and hearsay from the private sector providers. For quite some time, despite the changes in treatment policies, the IMCI guidelines have not been revised. They are still using the initial IMCI treatment charts which still refer to chloroquine as the first line treatment for malaria. They are so outdated. Yet our nursing assistants have no other reference materials to treat children other that the unrevised IMCI treatment charts (District Health Official, Jinja District).*

Due to resource constraints and limited opportunities to decide on procurement, the district officials argued that they did not control the provision of child–appropriate dosage formulations. They remain on the receiving end and accept whatever is distributed by NMS.

## Perspectives from the health workers

Health workers who participated in the interviews were differentiated by their level of training. Level of training influenced the health workers’ views about IMCI and child-appropriate dosage formulations. Health workers interviewed fall into two broad categories. The first includes pediatricians who are highly specialized health workers in diagnosing and treating children. Ideally pediatricians are supposed to handle referral cases from the lower level health workers from the outpatient department (in case of hospitals) and lower level health facilities. In the Ugandan public health facilities, pediatricians mainly work in hospitals where they are supposed to handle complicated cases. The second category included the rural based and less trained health workers who were mainly clinical officers, midwives, nurses and nursing assistants. Ideally, rural based health workers are supposed to treat less severe symptoms using IMCI and refer severe cases and any other complications to the pediatricians.

### Pediatricians’ perspectives and their practices

The general view from the pediatricians was that IMCI does not cater for child-friendly medicines due to lack of a conducive medicine policy environment. Pediatricians reported that they do not use IMCI approach because they are consultants for very sick children after their management using the IMCI has failed. It was therefore more practical to think of more advanced and international guidelines to diagnose complicated cases among children.

*For us we are beyond IMCI. We handle problems where IMCI has failed. We are consultants who are supposed to read widely. We refer to the internet, the British National Formulary and for that matter, we read beyond the Uganda Clinical Guidelines. However, because the clinical guidelines are not followed (by some of the lower level health workers) we often handle children with ordinary conditions and this increases our patient load (Pediatrician, Jinja regional referral hospital).*

Pediatrician supported provision of child-appropriate dosage formulations. From their own point of view, the best child-appropriate dosage formulations were mainly liquid oral dosage formulations (syrups). They were for example concerned about the government policy that phased out syrups that were being used for the treatment of pneumonia and malaria. They argued that some of the emerging complications and treatment failure were due to use of medicine formulations that were not appropriate for children.

*Some of the children referred to us from the lower level health facilities will have received the right treatment in terms of the medicines. However, because most of these will have been given in formulations of tablets, caretakers usually report difficulties in administration of the medicines. These difficulties result into inaccurate dosing sometimes associated with vomiting of the medicines. Caretakers do not know what to do when the child vomits. Most of them stop giving the medicines. We have been hearing these complaints from the caretakers quite a lot (Pediatrician Jinja Regional Referral Hospital).*

Pediatricians from the regional referral hospital and the national referral hospitals reported that the Uganda Clinical Guidelines (UCG) were insufficient for the management of the prevailing childhood illnesses. They therefore, did not necessarily prescribe medicines indicated in the UCG.

Pediatricians reported that they often prescribed medicines that were not in the public health facilities but were nevertheless considered to be child-appropriate by the pediatricians. When the medicines are not available, pediatricians said they referred caregivers to procure the medicines from the private pharmacies and private clinics where the medicines were available at a cost.

### Perspectives and practices rural based health workers

Rural based health workers reported (it was also observed) that they were still using the outdated IMCI treatment charts of 2001. They had not been oriented on the new treatment policies for quite some time and support supervision had accordingly diminished. It was noted that child-appropriate dosage formulations such granules, sprinkles and dispersible tablets were not mentioned among the key dosage formulations that health workers used in their daily practice using IMCI. They were instead familiar with tablets, suppositories for malaria and, injectables for pneumonia and sepsis.

Some of the Nurses and Nursing Assistants who had been in service for some years said they were initially trained in IMCI. However, they had also spent a long time without attending IMCI related refresher courses and felt that refresher trainings would have informed them more about new medicines, changes in treatment regimens, new guidelines and any other new information about managing childhood illnesses.

*We come to learn about these new medicines from our friends who work in private clinics. Some medicines are brought here for use when we have never heard about them. The IMCI charts you see hear are referring to very old medicines some of which have already been phased out. We are often confused by this (Nurse, rural based health facility).*

Some of the midwives who were interviewed reported that they had been left out during the initial IMCI training perhaps because it was never anticipated that they would ever interface with children. However, due to staff shortages, midwives were found treating children. Midwives noted that their work of treating children would be enhanced if they were also trained on how to use IMCI charts to treat children.

Health workers reported information gaps due to failure to update IMCI treatment charts to reflect medicines recommended by the new treatment policies. Much as the UCG had been revised from the 2007 to 2010, then later in 2012, only the in-charges in the rural based facilities had access to the revised guidelines. Health workers reported experiencing challenges regarding new treatment policies that were by then not integrated into the IMCI treatment charts. This, according to some, undermined their confidence in relation to deciding on which medicines to give. The newly introduced medicines for malaria were not written anywhere on the IMCI treatment charts that they were using. For example, while Artemether Lumefantrine was the first line treatment for malaria recommended in the new national malaria treatment policy, the IMCI treatment charts referred to a combination of fancidar and chloroquine-medicines which had been phased out by the National Drug Authority. Their other source of confusion was that the fansidar-chloroquine combination was also being recommended in the UCG 2010. At the same time, there were other types of anti-malarial medicines such as artesunate suppository and artesunate injectable. Despite the artesunate rectal and injectable formulations not being widely available in the health facilities, health workers often came across them either in the private sector or the community. Some interfaced with them because they were being channeled through their health facility to the community for the ICCM program. Although they were advised by their supervisors to use Artemether lumefantrine for malaria, health workers had also informally heard about use of rectal artesunate from the community based health workers. Although rectal artesunate (both injectable and suppository) was up to date and considered appropriate for children, they were often out of stock and health workers did not know when to use these two formulations. Some health workers still knew that chloroquine was still the main medicine for malaria. One of the Nursing Assistants innocently asked why chloroquine was no longer being supplied in the health facilities.

The health workers’ understanding of the new treatment protocols varied according to the level of training. Comparatively, senior health workers especially pediatricians were more conversant with the new treatment policies than the lower level health workers in the rural facilities who did not have sufficient information about new medicines for children. The missing interactions between the rural based health workers and their supervisors who included the district health teams and central level MoH staff partly explains the health workers’ limited understanding of new treatment policies. Although medicine policies for key causes of morbidity among children had been changing, the transfer of information about new policies from the global through the national level to the facilities had not been effective. Besides that, most of the rural based health workers such as Nursing Assistants said they did not have access to the revised Uganda Clinical Guidelines of 2010. In most of the facilities, it was reported (and also observed) that the guidelines were locked in cupboards of their supervisors who were often absent. The few lower cadre health workers who had glanced at the Uganda Clinical Guidelines of 2010 indicated that the guidelines were too bulky for them to use in their daily work and for that reason they had put them aside.

*These guidelines (Uganda Clinical Guidelines) are too big. You have to turn so many pages to get to the exact information about a disease. In addition to that, we have never been oriented on these guidelines. There are some technical terms which some of us cannot understand. We find the IMCI counseling and treatment charts easy to use. Unfortunately, these IMCI charts have outdated information where some of the medicines are no longer recommended (Nursing Aide rural based health facility Jinja district).*

On the other hand, the outdated IMCI treatment charts were visible with health workers who reported them to be more user friendly than the UCG. Some of the health workers wondered why medicines like the combination of chloroquine and SP which had already been phased out by the National Drug Authority were still appearing on the IMCI treatment charts. The same medicines were no longer being supplied by NMS and there were some health workers who did not know that chloroquine and SP had been phased out.

*You hear that the treatment of malaria has changed from chloroquine and SP to ACTs. Our (IMCI treatment) charts continue to indicate chloroquine and fansidar for children. By the way we no longer get chloroquine among the medicines supplied here. What is happening? (A Nursing Assistant rural based health facility).*

Some of the health workers were not sure of the first line treatment for pneumonia. They wanted to know whether amoxicillin and cotrimoxazole should be prescribed interchangeably. They said they were using both septrine and amoxicillin (depending on what was available at the time of dispensing) as the first line treatment for pneumonia. The child-appropriate amoxicillin dispersible tablet was still limited in supply and was mainly delivered for the donor funded ICCM program. Dispersible amoxicillin tablets were often withdrawn from the village health team providers for use in the facilities to avoid expiry especially because fewer children were being seen by the community providers. Cotrimoxazole tablets were on a high demand because it was also being used for HIV/AIDS prophylaxis.

*We are using both cotrimoxazole and amoxicillin as first line treatment for pneumonia depending on what is available in stock when the child is brought for treatment. In most cases, cotrimoxazole (septrine) is used because it is supplied more regularly. On the other hand, amoxicillin is rarely used because it is always out of stock. Much of what we see is a remnant of what was being taken to the community for ICCM. We even do not know the medicine which is recommended for pneumonia. We see some of the donors supplying amoxicillin to the community. On the other hand, government concentrates on the supply of cotrimoxazole. Sometimes we take the remainders of amoxicillin dispersible from because there are challenges of storing these medicines in the community for a long time. They can easily expire before use. In any case, we are seeing many more children here than they see in the community (Clinical officer, in-charge of a rural based health facility).*

Rural based health workers had limited knowledge about the globally recommended ‘child size’ medicines and child-appropriate dosage formulations concepts. There was a consistent affirmation by the health workers indicating that IMCI treatment charts and the clinical guidelines neither referred to ‘child-appropriate’ dosage formulations’ nor the ‘child size medicines’. In spite of this limitation, health workers presented informative viewpoints about the different dosage formulations they were using to treat children. Health workers in the rural areas highly commended IMCI as a suitable program for children but also hastened to add that the approach had flaws: lack of updated guidelines; missing support supervision and, lack of refresher trainings. They echoed lack of child-friendly medicines in the assortment of medicines supplied by NMS.

The rural based health-workers’ viewed appropriateness of dosage formulations in terms of being easy to prescribe, dispense and administer. In their practice, they came across ordinary tablets and capsules, oral suspension powders and a few dispersible tablets. They also reported using injectables and suppositories to treat children under severe conditions. From the experiences they shared, formulations in tablet forms and capsules were a challenge for children to swallow. Yet tablets and capsules were the most supplied by government for children. Rural based health workers also expressed their opinions about formulations which they though were child-friendly. These were discussed with reference to the common diseases of malaria and pneumonia. For malaria, health workers maintained that coartem (Artemether lumefantrine) was child-appropriate in terms strengths. They do not have to split a tablet of coartem even for an infant. However, like any other tablet, Artemether lumefantrine has to be crushed before being administered to the child. Artemether lumefantrine they were using was not dispersible in water like the ones that were being provided in the community and hence this type was diffecult for children to swallow. Therefore, rural based health workers said they have often advised caregivers to crush the tablets in order to make them suitable for children. Even when broken into pieces, some health workers noted that tablets were still difficult for children to swallow especially when the tablets are bitter. Regarding pneumonia medicines, health workers complained about lack of septrine syrup in the health facilities. The most available was the adult size of 480 mg. As a result, health workers said they were breaking the big tablets of septrine into smaller pieces before administering them to children. Despite amoxicillin being the recommended first line medicine for pneumonia, the child-appropriate dispersible amoxicillin tablets which were available in only a few of the health facilities as remnants of what was used in the community. Some of the health workers admitted having limited understanding of what dispersible tablets were and how they are used. Some were concerned because they did not see dispersible tablets being mentioned anywhere in the Uganda Clinical Guidelines and the IMCI charts they were using.

*We hear dispersible tablets being talked about, but we have not seen anywhere where they are written (Nursing Assistant, rural based health facility)*

Perhaps due to limited knowledge and limited publicity, dispersible tablets were not considered as the most important as syrups, suppositories, injectables and ordinary tablets.

There were concerns that liquid oral formulations commonly referred to as syrups were missing and yet according to the health workers they are the best for children. The common “syrups” that the health workers had heard about were for septrine, amoxicillin and chloroquine. There were a few rural based health workers who were critical of syrups and said that they were problematic because they were too expensive and difficult to store and that was the reason why they were not being procured by government.

*Sometimes there are some medicines whose consumption is low. In such cases, these are challenges of storage which makes the medicine susceptible to being damaged if they are syrups (Nurse, a rural based health facility).*

The most commonly mentioned liquid oral formulations were metronidazole syrup used to treat diarrhea and nevirapine syrup for prevention of mother to child transmission. Metronidazole syrup was being supplied in all the health facilities. Health workers wondered why metronidazole syrup was regularly supplied in most of the health facilities and yet other important syrups for malaria and pneumonia were not provided. Although they had been deleted from the EMHSLU, syrups for septrine and amoxicillin were still perceived by health workers as critically needed for treating children and the concern was that the only available formulation for septrine was the tablet of 480 mg tablets meant for adults as earlier discussed. The available Amoxicillin capsules were perceived to be unsuitable for children.

*Ok, I say syrups are expensive fine: but they should add in a few syrups especially antibiotics such as septrine and amoxicillin so that we can continue with them. We only have septrine tablets of 480 mg which are too big for these children. I think it can help if there is septrine syrup instead. The rest of the medicines can remain as tablets (a Nurse in a rural based health facility).*

Injections were reported to be highly available and highly utilized for treating children especially for severe pneumonia conditions. Injections were rated as very important especially by the rural based health workers and were being used when ‘syrups’ were not available  and were preferred for those children who vomit oral medicines.

*For pneumonia, we always have problems with the medicines in stock. We mainly have septrine which is too big for the child to swallow. Septrine syrup would be ideal but currently syrups are not being provided by government and we do not know why. We also have amoxicillin capsules which are the most available but they are difficult for children to swallow and they are meant for adults. Therefore, for pneumonia, we often use injection of x-pen which is not allowed, we just need to use it for pre-referral treatment. We are forced to use it especially when the child is vomiting (Nursing Assistant in a rural based facility).*

In a nutshell, rural based health workers persistently emphasized the fact that, the treatment guidelines and the IMCI treatment charts were not clear about child-appropriate formulations. Despite being the main approach used to diagnose and treat children, health workers rightly argued that IMCI had nothing to do with chid-appropriate dosage formulations. Only names of medicines were simply referred to as oral, injectable and suppository. Categories of oral medicines formulations which are child-friendly are not referred to in the guidelines.

## Discussion

The overarching assumption for the WHA resolution of ‘better medicine for children’ was that beneficiary countries would adopt this recommendation into their policy and implementation frameworks. The study design was based on the premise that child-appropriate dosage formulations are the best option to improve prescription, dispensing and administration of medicines to the under-five population. Findings from the qualitative inquiry at the ministry; district and facility levels established that there has been no integration of child-appropriate dosage formulations into the IMCI approach in Uganda. From the perspectives of the respondents at the three levels, 3 related explanations for failure to integrate are apparent: (1) resource constraints that affected persistent implementation of the IMCI program (2) gaps in the global and national efforts to integrate child-appropriate dosage into the Ugandan essential medicines list (3) cognitive gaps and information distortions that have undermined health workers’ confidence in prescribing, dispensing and administering medicines to children while using outdated IMCI treatment charts.

### Resource constraints and persistent implementation of IMCI

Resource constraints were constantly referred to in the perspectives that were explored at the national, district and facility levels. Resource constraints question can be viewed in two dimensions. The first dimension was that whereas IMCI is the main approach used to manage childhood illnesses in Uganda, it has not been sufficiently funded to address ever-changing global policy prescriptions such as the integration of chid-appropriate dosage formulations. In reference to the theoretical framework, resource constraint barrier was highlighted as a barriers by Tansella &Thornicroft (2009) in their three phases of implementation model for evidence based clinical practices [12].

Due to resource constraints, MoH could not mutually adjust the health system to meet the new global demand of child-appropriate dosage formulations. Therefore, the recommendation for ‘better medicines for children’ came at a time when resources for supporting IMCI had already dwindled. Lack of sustainable funding for IMCI undermined its effectiveness in terms of the integration of child-appropriate dosage formulations.

The second dimension was that right from its inception, the recommendation for ‘better medicines for children’ later coined as ‘child-appropriate dosage formulations’, did not receive specific donor attention. Except for the Melinda Bill gates Foundation which facilitated piloting of the child size medicine in three countries of Ghana, India and Tanzania, there were no other global efforts to market the concepts of child-appropriate dosage formulations. The idea of child-appropriate formulations remained in the UN (UNICEF and WHO) circles for quite long and did not gain global prominence until the UN declaration of the ‘essential commodities and lifesaving priority medicines for children’.

Dependence on external funding for critical programs such as IMCI has done a disservice in some aspects. Interestingly, bypassing the national systems, largely ignoring the existing policies and medicine supply systems, development partners in Uganda initiated and supported supply of child-appropriate dosage formulations to the community [7]. Although this marked the beginning of the era of child-appropriate dosage formulations in the country, failure to mainstream the support through IMCI the main approach for treating children and due to failure to revise policy provisions to reflect child-appropriate dosage formulations [8], the initiative from the development partners left a lot desired. The availability and utilization of child-appropriate lifesaving and priority medicines for children continued to remain low in public facilities [9]. In the same way, due to lack of funding, the sustainability of IMCI, which was initially a heavily donor funded program, became uncertain. The findings of this study support the argument of Tansella and Thornicroft that resources are critical even during the final phases of projects [12]. Similarly, Hogwood and Gunn (1984) argued that external circumstances to the implementing agency may impose crippling effects if there is no adequate time and resources for continuity [13].

At the time when resources were scarce MoH decentralized some of the key IMCI activities such as refresher training, support supervision to the district without decentralizing commensurate resources. It is not surprising therefore that these activities were not implemented by the district. According to Walt (1994), decentralization of responsibilities without the associated resource requirements may make local authorities vulnerable to policies which are not cost effective. On the other hand, decentralization of financial resources can help enforce the guidelines and other elements of the packages such as support supervision and refresher courses [14].

The success of the integration of child-appropriate dosage formulations will largely depend on the capacity of the health system to enforce compliance. Health system strengthening was anticipated as one of the fundamental elements of the IMCI strategy [21, 22]. This study supports the argument that dynamic and strong health systems would enable the system to achieve improved health outcomes [23]. In Uganda, there have been more investments in the ICCM with diminishing support for another equally important component, the facility based IMCI [7]. Notwithstanding the fact that the facility based IMCI and ICCM should be well integrated if it is to have an impact on mortality. In Uganda, as a result of this gap, the expected efficiency and effectiveness of the IMCI are not being much felt. While the community-based initiatives extend the delivery of interventions in remote areas where health services are hard to access, strengthening national health systems should be the long-term aim [24]. Health systems bottlenecks are a formidable challenge in the rural based health facilities in Uganda and this directly affects integrating child-appropriate dosage formulations. As a matter of fact, the health systems' gaps in Uganda who include lowly trained health personnel who have not been oriented about new medicines, whose IMCI treatment charts are outdated and in the context of limited supply of child-appropriate dosage formulations and challenges of referral explain the failure to easily integrate child-appropriate dosage formulations into IMCI. A study elsewhere shows a similar pattern regarding IMCI implementation whereby the workload, medicine supplies and poor supervisory mechanisms undermine the program [25].

Due to resource constraints, IMCI remains an unfinished business in the clinical practice in Uganda. Tansella & Thornicroft (2009) highlight the need for sustainable: advocacy, financial support, and continuous staff training and review of the information technology. This did not happen in the case of Uganda because IMCI was funded in the initial stages by the donors and this funding stopped later. Government was unable to sustain all the IMCI core activities that were initially supported by donors. In the end, the globally generated seeds of child-appropriate dosage formulations landed on the bare ground since IMCI as a key framework for treating children was not adequately funded. Tansella and Thornicroft (2009) in their framework about translation of evidence into practice have contended that the greatest impact of a policy is not adoption per se but rather from the long-term implementation of the practice where resources are key [12]. Hogwood and Gunn (1984) added that the combination of resources is a must for the implementation to take place [13].

### Lack of global and national effort

MoH officials attributed lack of integration of child-appropriate dosage formulations to gaps in the global and national effort. Although the IMCI algorithm at the global was adjusted by WHO to take care of ‘better medicines for children’, this did not happen at the national level in Uganda. There were no official discussions involving recipient countries and WHO about how to introduce child size medicines in Uganda [7]. It has been reported that changes in Malaria treatment policies were largely influenced by WHO active involvement in financing and initiating local discussions about the new policies [26]. For the integration to take root, it would have been necessary at the national level to develop a policy framework for integrating these medicines into the essential medicines list and clinical guidelines. Macleod Stuart and colleagues (2013) argue that it is necessary to update clinical guidelines, train health workers accordingly about the need to integrate child-appropriate dosage formulations into the essential medicines lists and the standard treatment guidelines which would guarantee knowledge transfer and dissemination of the new medicine information [27]. The disarticulation between IMCI approach in Uganda and the global recommendations that emphasize child-appropriate medicine formulations [1, 11] was apparent. This disarticulation has been due to lack of specific policy focus on child-size medicines both in the essential medicines lists and the Uganda clinical guidelines (UCG) [8]. The Uganda government failed to formally adopt the global policy on child-appropriate dosage formulations [7]. Eventually this limited the avenues for IMCI to address the global requirement of integrating child-appropriate dosage formulations. For example, the Uganda Clinical Guidelines of 2010 and 2012 were silent about child-appropriate dosage formulations.

There was concern that quality and effective of treatment of children less than 6 years of age cannot be realized without integrating the child-appropriate dosage formulations into the essential medicines lists. Worldwide essential medicines lists are considered vital for prioritizing medicines to procure. Holloway and Henry have shown that essential medicines along with training on standard treatment guidelines are associated with quality of medicine use in many countries [28]. In Uganda, child-appropriate dosage formulations are not among the essential medicines and worst still, health workers are not regularly oriented about the global changes in the medicines for children. Similarly, in South Africa health workers found training in IMCI informative and reportedly increased their skills for managing the sick children. Follow up visits would help encourage correct practice and motivate the newly trained IMCI practitioners [29].

### The cognitive gap among health workers

Pediatricians were more confident about the Uganda national treatment policies of the key childhood illnesses and were also able to access state of art international standards for the management of childhood illnesses. However, pediatricians are very few and are mainly available in the hospitals. On the contrary, rural based lower cadre health workers such as Nursing Assistants did not have sufficient information about new treatment protocols. This information gap was associated with lack of revised guidelines, lack of refresher training and gaps in information mainstreaming when new treatment protocols are introduced through vertical programs such as the presidential malaria initiative. In this initiative, new medicines for treating malaria were recommended but were not reflected in the IMCI treatment charts and clinical guidelines. Medicines recommended in the clinical guidelines as first line treatment for malaria and pneumonia among under-five were outdated medicines which were also not even child-friendly [8]. At the same time, the new malaria treatment policies proposed through the vertical program of presidential malaria initiative were not effectively communicated to the lower cadre staff in rural based facilities. This is because of the limited integration of these vertical programs into the general health care delivery systems such as IMCI. Unless the issue of new knowledge is addressed in the IMCI approach to take care of evidence based medicines and new approaches to management of childhood illnesses, IMCI will eventually become irrelevant and will be taken over by vertical programs such as ICCM and malaria programs as has been the case before. Innovative solutions may be necessary to ensure that guidelines are updated to reflect child-appropriate medicines and at the same time health workers’ skills should be updated and maintained [29].

Lack of information affects easy understanding of new developments about medicine policies. Okungu and Gilson (2014) argued that effective health policy communication is important for the uptake of new drug interventions and adherence to treatment regimens [30]. There was confusion about first line medicines due to information gap resulting from the failure to update the IMCI treatment guidelines, missing refresher courses and limited support supervision. It has been argued that development and implementation of (evidence-based) clinical practice guidelines is one of the promising and effective tools for improving the quality of care for children [31] especially when introduced in the context of rigorous evaluations and subsequent updates [32]. Some of the good attributes of clinical guidelines include an explicit description of the scientific evidence for which the recommendation is available; evidence that is straightforward and not conflicting; and when the evidence is based on relevant aspects of care in daily practice [33]. At the global level, the most recent version of IMCI guidelines was produced in 2014. Nevertheless, the new version is still vague about child-appropriate formulations. For example, in the UCG there are recommendations such as ‘give oral amoxicillin for 5 days’ on page 2 of the UCG or ‘give first line oral antimalarial’ on page 4 of UCG [34]. The IMCI treatment charts that were being used in all the rural based health facilities by the time of the study had not been updated since 2001. This explains the confusions and lack of confidence especially among the lower level rural based health workers.

### Policy implications

According to the Health Sector Strategic and Investment Plan III, IMCI remains the main approach in the management of childhood illnesses in Uganda [6]. In reality, IMCI is the most appropriate approach for the rural based health facilities where children are treated by low cadre health workers. Therefore, IMCI should constantly receive adequate attention during the policy development, planning, budgeting and implementation processes. Without sufficient investments into the IMCI core activities, the expected gains from IMCI will not be achieved. Core investments should include regular adaptation of new generic guidelines from WHO, sufficient facilitation in terms of training, support supervision and regular to ensure that there is an environment in which health workers can apply their child care skills [35]. It also means that all the vertical programs such as the presidential malaria initiative and ICCM should be aligned with and integrated into the IMCI program whereby guidelines of these vertical programs are part and parcel of the IMCI treatment charts. Pediatricians continue to prescribe liquid oral formulation (syrups) which are not readily available in the public facilities and caretakers have to purchase them at high costs. The implication is that some of the caregivers who cannot afford the medicines might have to forego the treatment or procure under dose because they cannot afford. This infringes on the rights of the poor to health services.

## Conclusion

The IMCI package for managing childhood illnesses in Uganda does not include the global recommendation of ‘better medicines for children’. The poorly funded IMCI strategy has continued to stagnate due to lack of resources for updating the guidelines, procure evidence based medicines, conduct refresher training and support supervision for health workers. This stagnation continues to undermine appropriate and effective administration of medicines to children. The missing child-appropriate dosage formulations in the EMHSLU and UCG undermine low cadre rural based health workers efforts to appropriately treat children using the IMCI approach. There are still challenges in prescribing, dispensing and administration of tablets and capsules to children under-five. Depending on their level of medical training, health workers have come up with strategies for mitigating the challenges of administration of adult medicines to children. Lower cadre health workers prefer using injections and suppositories which they more appropriate for children who vomit. When revising the clinical guideline, it is important to seek the views of those providers managing children especially lower cadre staff who have narrow choices of medicines, have limited information about new medicnes and are also faced with challenges of medicine formulations that are inappropriate for children. 

### Recommendations

Both government and development partners should invest more into the IMCI strategy from a health systems approach to enable the systems to respond positively to the emerging global demands for child-appropriate dosage formulations. This would require more budget allocations to the child health division and other relevant departments. The IMCI treatment charts should be updated regularly to reflect child-appropriate dosage formulations. It will also necessitate revisiting the EMHSLU to reflect child-appropriate dosage formulations based on new evidence. There should be adequate budget provisions for refresher training and support supervision for the health workers on new evidence-based and national treatment policies for the common childhood ailments. More studies are needed to explore the experiences of the caregivers about access and use of child-appropriate dosage formulations. 
